# Occurrence of macrophyte monocultures in drainage ditches relates to phosphorus in both sediment and water

**DOI:** 10.1186/2193-1801-2-564

**Published:** 2013-10-25

**Authors:** Jeroen P van Zuidam, Edwin THM Peeters

**Affiliations:** Aquatic Ecology and Water Quality Management Group, Wageningen University, PO Box 47, 6700 AA Wageningen, The Netherlands; Ecology & Biodiversity Group, Utrecht University, PO Box 80.058, 3508 TB Utrecht, The Netherlands

**Keywords:** Diversity, *Elodea nuttallii*, Eutrophication, Duckweed, Monoculture, Standing stock

## Abstract

Monocultures of functional equivalent species often negatively affect nutrient cycling and overall biodiversity of aquatic ecosystems. The importance of water and sediment nutrients for the occurrence of monocultures was analysed using field data from drainage ditches. Ranges of nutrients were identified that best explained the occurrence of monocultures of *Elodea nuttallii* (Planch.) St. John (Waterweed type), monocultures of duckweed (Duckweed type) and the occurrence of a diverse submerged vegetation (Mixed type). Results indicated these three vegetation types occurred at distinctive ranges of phosphorus in water and sediment. Sediment phosphorus distinguished monocultures from the Mixed type, with the two monocultures occurring at two to four times higher concentrations. The Waterweed type occurred at higher sediment phosphorus levels than the mixed type, showed a higher degree of dominance and lower number of red list species. Phosphorus concentrations in water were four to six times higher in the Duckweed type compared to the Waterweed and Mixed type. The three vegetation types had comparable total biomass which was unexpected. This comparability was likely caused by duckweed only growing at the water surface at the highest nutrient levels and the limited space in drainage ditches for increased submerged biomass development at high nutrient availability. Possible measures to limit the occurrence of monocultures, and thereby increasing the ecological quality, are discussed with focus on lowering phosphorus concentrations in both water and sediment and on removal of plant species that develop into monocultures.

**Electronic supplementary material:**

The online version of this article (doi:10.1186/2193-1801-2-564) contains supplementary material, which is available to authorized users.

## Introduction

Environmental change in ecosystems due to anthropogenic stressors generally leads to a loss of community diversity and is often combined with an increased dominance of one or a few species (Hillebrand et al. 2008). An aquatic vegetation dominated by for instance several duckweed species can be considered a monoculture as the species are functionally comparable and therefore influence the ecosystem in a comparable way (Hubbell 2005). Most duckweed species are fast growing, disturbance tolerant plants that can contribute to low light availability, anoxia and a loss of biodiversity at high coverage (Janse and Van Puijenbroek 1998). Since monocultures might be difficult to mitigate due to their resilience (Scheffer et al. 2001), they often cause undesired changes in ecosystem functioning. In Argentinian grasslands, dominant exotic plant species may produce easier decomposable litter, resulting in increased soil decomposer activity, supporting continued dominance of the exotic species (Spirito et al. 2012). In wetlands, the dominance of *Juncus* species may lead to altered light conditions, negatively affecting species richness and abundance (Ervin and Wetzel 2002).

In aquatic systems, similar negative effects of dominance are found. In lakes for example, eutrophication and related phytoplankton dominance prevents submerged macrophyte development, which leads to an overall loss of biodiversity (Scheffer et al. 1993). Alternatively, small aquatic systems such as streams and drainage ditches can develop monocultures of fast growing submerged species like *Elodea nuttallii* (Planch.) St. John or duckweed (Peeters et al. 2013) which can affect nutrient cycling and plant diversity (Janse and Van Puijenbroek 1998; Di Nino et al. 2005).

Changes in species composition of aquatic systems often result from increased productivity following elevated nutrient availabilities (Squires and Lesack 2003). Oligo- to mesotrophic lakes and ditches contain a well-developed vegetation with different submerged, mainly annual plant species (Forest 1977; Netten et al. 2010), but also *Elodea nuttallii* may be found at low coverages (Nagasaka 2004). Standing biomass typically ranges from 100-150 g DW/m^2^ or lower in oligotrophic conditions (Downing and Anderson 1985; Bloemendaal and Roelofs 1988; Nagasaka 2004) to 200-400 g DW/m^2^ at mesotrophic conditions (Forest 1977;van Wijk 1988). At eutrophic conditions the submerged vegetation may become dominated by a monoculture of fast growing, evergreen submerged species like *E. nuttallii* that, under these nutrient rich conditions, outcompete other submerged plants (Portielje and Roijackers 1995; Kadono 2004; Arts and Leenders 2006) with a standing biomass between 500 – 1000 g DW/m^2^ (Bloemendaal and Roelofs 1988; Ozimek et al. 1990; Di Nino et al. 2005). At very high nutrient inputs (for instance 88 gr N m^-2^ year-1 and 14 gr P m^-2^ year^-1^ (Janse and Van Puijenbroek 1998)) a duckweed dominated vegetation may develop in shallow and sheltered aquatic systems with a standing biomass of 100-200 g DW/m^2^ (Bloemendaal and Roelofs 1988; Janse and Van Puijenbroek 1998;2005).

Dominance of *Elodea* or duckweeds is frequently found in drainage ditches in the Netherlands as well as the diverse vegetation composition (Netten et al. 2010). Studies analysing relationships between macrophyte species composition and nutrient concentrations mainly focus on large aquatic systems (e.g. lakes) and often only take into account one source of nutrients (either water or sediment). It appeared that in those large aquatic systems, vegetation composition is influenced both by water and sediment nutrient levels (2003; James et al. 2005; Sayer et al. 2010). In smaller sized aquatic ecosystems such as drainage ditches, a positive relation was found between phosphorus (P) loading and the degree of duckweed dominance in early successional stages of vegetation development in experimental drainage ditches (Portielje and Roijackers 1995). Furthermore, a shift from submerged plants to duckweed dominance could be modelled through increased nitrogen (N) levels in water (Janse and Van Puijenbroek 1998; Scheffer et al. 2003). Van Liere et al. (2007) showed that critical nutrient levels at which those shifts may occur were 0.19-0.42 mg P/L and 1.3-3.3 mg N/L. Although there is general consensus that nutrients play a crucial role in changing plant community compositions of aquatic systems (Carpenter et al. 1998), few field studies have investigated the importance of nutrients from both water and sediment for the occurrence of monocultures in small aquatic systems. The present study therefore relates both water and sediment nutrient concentrations to the occurrence of a diverse submerged vegetation type and two frequently observed monocultures in drainage ditches. Vegetation types are characterized by their biomass, since biomass is one of the direct results of nutrient availability. The central question of this study is which nutrients in water and sediment correlate best with differences in biomass composition of drainage ditch vegetation. The objectives are to (1) identify nutrient fractions in water and sediment that best explain the occurrence of monocultures (of *E. nuttallii* or duckweed) and diverse submerged vegetation and (2) determine the nutrient ranges at which the three vegetation compositions occur.

## Materials and methods

### Drainage ditch characteristics

Ditches in the Netherlands are mainly found in agricultural areas. Average width of these ditches is around 4 m and depth is around 50 cm with water levels being mostly constant (field observations from this study). Generally both duckweed and submerged plants (such as *E. nuttallii* and *Potamogeton* sp.) occur throughout the ditch profile. In the Netherlands, plant growth shows a seasonal pattern starting around April and reaching peak biomass around the end of August. During autumn and winter, senescence of most biomass occurs (except for evergreen species). Ditches are usually mown yearly in autumn using a mowing bucket while dredging is performed once every 5-10 years.

### Data collection

Based on knowledge of regional water boards 50 ditches across the Netherlands known to be dominated by duckweed or by *E. nuttallii* or with a mixed vegetation in the previous five years were selected for the study. Field measurements were performed in June and September 2007 including sampling of water, biomass and determining species composition. Sediment samples were taken once in June (for descriptives see Table 1). Vegetation recordings were made with Tansley coverage classes (Tansley 1946) by selecting a part of the ditch, approximately 25 m long, representing the vegetation composition in the ditch. All present vascular plant species were collected by wading through the transect and were identified up to species level. Biomass samples were taken in the same 25 m section. Biomass samples of complete plants (roots and shoots) were taken by selecting 1-3 patches in the ditch that together covered all variation in species composition and coverage. Vegetation was collected from a surface area of 900 cm^2^. Total coverage of each patch in the ditch was used to calculate a weighted total amount of biomass per m^2^. Biomass of duckweed, *E. nuttallii* and other submerged plants was separated from each other in the field. Biomass of each of these subsamples was determined after removing any attached periphyton and sediment by rinsing with water and removing attached water by spinning the biomass around in a salad spinner for thirty seconds. To compare measured biomasses with those in literature, dry weights were estimated as being 10% of fresh weight (Forest 1977; Hasan and Chakrabarti 2009). Water samples were taken both in June and September. Three samples of the upper 20 cm of the water column were taken with a tube sampler, evenly distributed across the same transect in which vegetation data was collected and mixed into one homogenized sample (volume 1 L). From this sample, two subsamples of 50 ml were used for nutrient analyses of which one was directly filtered with a 0.45 μm filter to determine dissolved nutrients. Sediment was collected by taking a subsample from a homogenized sample consisting of three sediment cores, taken from the top 5 cm of the sediment along the transect used for collecting vegetation data. Water and sediment samples were stored in a freezer at -20°C directly after collecting and analysed within 2 months.

**Table 1:** **Distribution of the three biomass fractions (g fresh weight/m** ^**2**^ **) for the defined vegetation types**

		Duckweed type (n=20)	Waterweed type (n=17)	Mixed type (n=13)
**June 2007**				
Free floating plants	**Mean**	**1080**	**27**	**307**
	Standard error	240	15	155
	Minimum	14	0	0
	Maximum	3411	231	2056
*Elodea nuttallii*	**Mean**	**16**	**1102**	**233**
	Standard error	16	302	104
	Minimum	0	18	0
	Maximum	318	4708	1078
Other submerged plants	**Mean**	**0**	**91**	**794**
	Standard error	0	58	317
	Minimum	0	0	0
	Maximum	3	833	3444
Total	**Mean**	**1096**	**1219**	**1335**
	Standard error	239	320	319
	Minimum	14	18	150
	Maximum	3411	4708	3444
**September 2007**				
Free floating plants	**Mean**	**1120**	**61**	**202**
	Standard error	224	38	84
	Minimum	7	0	0
	Maximum	3811	572	866
*Elodea nuttallii*	**Mean**	**15**	**757**	**149**
	Standard error	15	149	60
	Minimum	0	11	0
	Maximum	300	2328	607
Other submerged plants	**Mean**	**11**	**43**	**1174**
	Standard error	11	29	336
	Minimum	0	0	67
	Maximum	210	502	4579
Total	**Mean**	**1146**	**860**	**1525**
	Standard error	221	165	376
	Minimum	7	11	79
	Maximum	3811	2385	4676

### Chemical analysis

Water samples were analyzed for nutrients using a continuous flow analyser (Skalar Analytical BV, Breda, The Netherlands). Total P, total N, orthophosphate (PO_4_^3-^) and dissolved N (nitrate (NO_3_^-^) and nitrite (NO_2_^-^)) were analyzed following standard protocols (NNI 1986; NNI 1990; NNI 1997). NO_3_^-^ + NO_2_^-^ and PO_4_^3-^ were determined using the Griess-Ilosvay reagent (NO_3_^-^ + NO_2_^-^ analysis) and ascorbic acid/antimony (PO_4_^3-^ analysis). Total N and P were determined after a UV/per sulphate destruction. Concentrations of total N and P in sediment were determined on a segmented flow analyser after destruction with sulphuric acid/salicylic acid/ selenium/ hydrogen peroxide, with total N measurement based on the Berthelot reaction and total P measured as phosphate molybdenum (Novozamsky et al. 1983; Novozamsky et al. 1984).

### Vegetation pre-analysis

**Figure 1:**
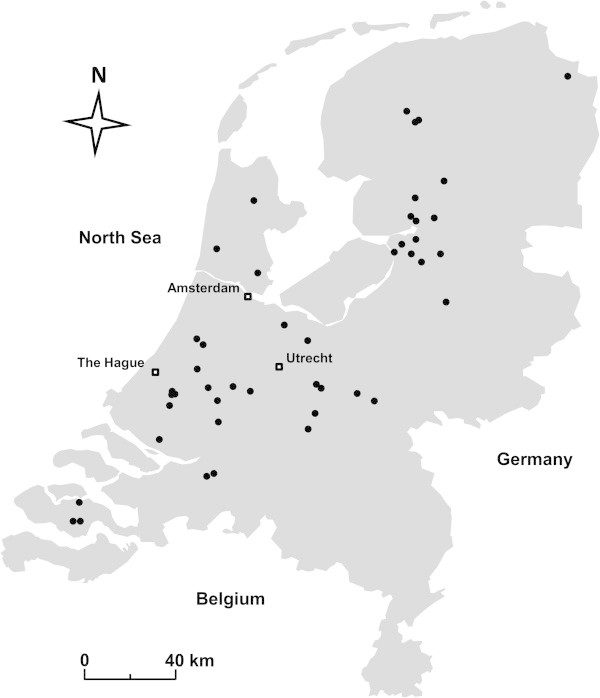
**Location of the sampled drainage ditches in the Netherlands (n=50).**

Data on the 50 ditches was used to appoint ditches to one of the three vegetation types; dominated by *E. nuttallii* (hereafter called *Waterweed type)* or duckweed *(Duckweed type)* or being diverse *(Mixed type*.) This was done by evaluating the amounts of the three sampled biomass fractions relative to the total biomass. Similar to Tansley’s coverage classes 8 and 9, indicated as co-dominance with coverage ranging from 50 to 100% (Tansley 1946), a ditch was labelled as Duckweed or Waterweed when more than 50% of the total biomass was made up of duckweed or *E. nuttallii,* respectively. It was assumed that Mixed type ditches contained biomass of a mixture of several species but with lower biomass of the ones responsible for monocultures. To this end, ditches were selected that showed more than 50% submerged biomass other than *E. nuttallii*. To be included in the study, ditches had to meet these criteria at least at the peak of the growing season (September), when the vegetation was fully developed. Figure 1 shows the distribution in the Netherlands of the 50 ditches involved in the data analysis described below.

Diversity measures and the number of red list species (species that are sensitive to disturbances and have decreased considerably in the last decades (LNV 2004)) were calculated for each vegetation type. Alpha diversity (diversity within a habitat) was calculated as the mean number of species per vegetation type. This was calculated as the total number of unique observed species in June and September per ditch, reflecting all species that can occur in the ditch throughout the growing season. Gamma diversity (total diversity on the landscape level) was calculated as the total number of species found in all ditches within each vegetation type. Beta diversity (the degree of differences in diversity between habitats) was calculated as Whittaker’s measure β_w_ = (γ/α)-1 (Magurran 1988). The degree of dominance in the vegetation composition was calculated as 1- Simpson’s index (D) with;

where n_*i*_ is the coverage of taxon *i* and n is the total macrophyte coverage in a ditch (Magurran 1988). The index ranges from 0 (all taxa are equally present) to 1 (one taxon dominates the community).

### Data analysis

Vegetation types were analysed for differences in nutrient concentrations, total biomass and diversity measures (α-diversity and dominance index) by performing Kruskal-Wallis tests with posthoc comparisons of vegetation types (Bonferroni corrected). Kruskal-Wallis tests were used due to non-homogeneous variances. Patterns arising from statistical analyses on data from the first and second sampling round were similar and therefore only results from the first round are presented. A Pearson’s correlation matrix was used to identify strongly correlated nutrient fractions. A multinomial logistic regression was done with the nutrient fractions that differed significantly (according to the Kruskal-Wallis test) and were not strongly correlated (according to the Pearson correlation) to determine the contribution of the various nutrient fractions in explaining the differences between vegetation types. To determine at what ranges of nutrient concentrations the vegetation types occur, 95% confidence intervals were calculated per vegetation type for the nutrient fractions that contributed most in the multinomial logistic regression. To test whether the three vegetation types occurred at significantly different combinations of water and sediment P concentrations, the level of group separation was tested with an ANOSIM with the Bray-Curtis distance measure and 10000 permutations, using PAST 1.91 (Hammer et al. 2001). All other statistical analyses were done using SPSS 18 (IBM 2009).

## Results

### Characteristics of vegetation types

Based on biomass composition, 20, 17 and 13 ditches were designated as Duckweed, Waterweed and Mixed, respectively. Both in June and September the Duckweed and Waterweed ditches contained the highest biomass of duckweed and *E. nuttallii* respectively, while Mixed ditches contained the highest biomass of plants other than *E. nuttallii* or duckweed (Table 1). Duckweed and Waterweed ditches contained on average 97% (S.E.= 1.5) duckweed biomass and 91% (S.E.= 2.3) *Elodea nuttallii* biomass, respectively, in both sampling rounds. The Mixed type contained on average 61% (S.E.= 6.6) submerged biomass (other than *E. nuttallii*), while average biomass of the other two biomass fractions was 19% (S.E. = 3.7) of the total biomass. Total biomass was comparable between the vegetation types for both months (Kruskal-Wallis, χ^2^=0.682, df=2, p=0.711).

Duckweed type ditches showed a lower α-diversity compared to the Mixed type ditches (Table 2, Kruskal-Wallis, χ^2^=10.132, df=2, p=0.006), but was comparable to the Waterweed type (for observed species see Additional file 1). The dominance index was higher for the Waterweed type than for the Duckweed and Mixed type (Kruskal-Wallis χ^2^=6.839, df=2, p=0.033) indicating a higher degree of dominance in the species composition of Waterweed dominated ditches. Both beta and gamma diversity were lowest for the Duckweed type and highest for the Mixed type. Additionally, from the total of five recorded red list species, none were found in the Duckweed type, only one species was found in the Waterweed type, while five out of thirteen ditches within the Mixed type contained one or more (maximum four) red list species.

**Table 2:** **Diversity measures for the three vegetation types**

Diversity measure	Duckweed type (n=20)	Waterweed type (n=17)	Mixed type (n=13)
α-diversity (number of species per ditch)	6.65 (0.65)^a^	8.53 (0.55)^a,b^	9.46 (0.69)^b^
β-diversity (γ/α-1)	2.46	2.52	2.91
γ-diversity (total nr. of species in vegetation type)	23	30	37
Dominance (1-Simpson’s index (D))	0.38 (0.04)^a^	0.52 (0.05)^b^	0.36 (0.04)^a^
Number of red list species	0	1	5
Number of ditches containing red list species	0	1	5

For α-diversity and Dominance mean values are shown with standard errors in parentheses. Letter codes (a,b) in superscript indicate the subgroup to which the vegetation types belong according to the Kruskal-Wallis posthoc comparison. For test statistics see results section. Dominance calculation is described in the methods section.

### Differences in nutrient concentrations between vegetation types

**Figure 2:**
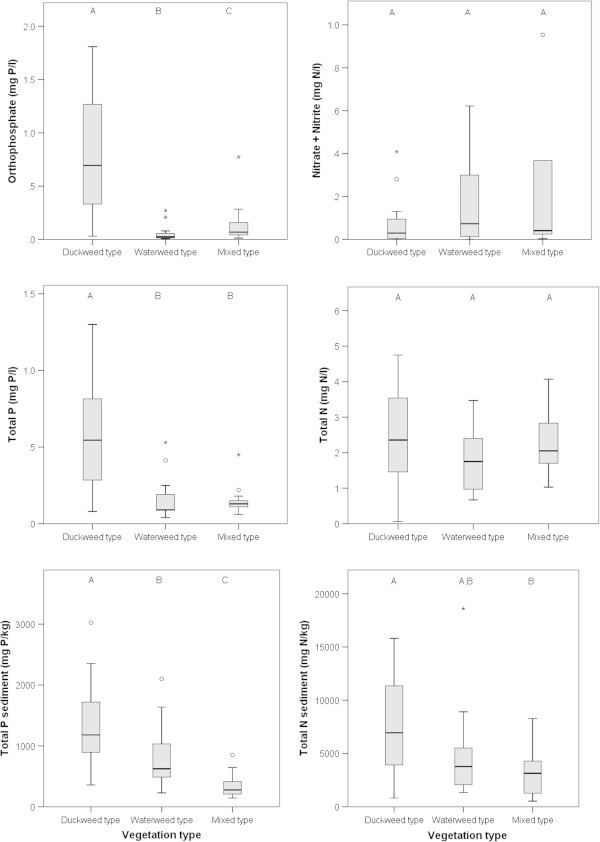
**Boxplots with ranges of nutrient concentrations of the three vegetation types, measured in June 2007.** The horizontal line within the grey box represents the median value. Grey box contains 50% of all values. Whiskers contain 75% of all values. Circles indicate outliers, asterisks indicate extremes. Letter codes on top of the boxplots indicate similar or significantly different groups according to Kruskal-Wallis tests with posthoc comparisons (Bonferroni corrected). For test statistics see Results.

Figure 2 shows that the Duckweed type was found at PO_4_^3-^ concentrations in water around ten to thirty times higher than the Waterweed type and the Mixed type (Kruskal-Wallis, χ^2^=28.597, df=2, p<0.001), whereas total P concentrations in water were four to six times higher in the Duckweed type than in the Waterweed and Mixed type (Kruskal-Wallis, χ^2^=23.760, df=2, p<0.001). Total P concentrations in the sediment were almost two times higher in the Duckweed type than in the Waterweed type, while concentrations in the Waterweed type were also around two times higher than those in the Mixed type (Kruskal-Wallis, χ^2^=22.112, df=2, p<0.001). NO_3_^-^ + NO_2_^-^ and total N concentrations in water did not show any significant differences (NO_3_^-^ + NO_2_^-^ : Kruskal-Wallis, χ^2^=2.473, df=2, p=0.290; total N: Kruskal-Wallis, χ^2^=2.873, df=2, p=0.238), while total N in sediment differed between the Duckweed and Mixed type. Total N sediment concentrations in the Waterweed type were comparable to both the Duckweed and Mixed type (Kruskal-Wallis, χ^2^=8.823, df=2, p=0.012).

Weak correlations were found between total N - NO_3_^-^ + NO_2_^-^ in water, total P sediment – total P water and total P sediment – total N sediment, values ranging between 0.450 and 0.500 (Table 3). A moderately high correlation was found for PO_4_^3-^ and total P in water. Therefore, the multinomial logistic regression was done with total P in water and sediment and total N in sediment as explaining variables. The resulting regression model explained 45% more of the variance compared to the model with only the intercept (χ^2^=48.769, df=6, p<0.001). Total P in water (χ^2^=20.255, df=2, p<0.001) and total P in sediment (χ^2^=12.039, df=2, p=0.002) contributed significantly to this explained variance with net contributions of respectively 41.5% and 24.7%. Total N in sediment contributed 2.6%, which was not significant (χ^2^=1.288, df=2, p=0.525). Therefore total P in water and sediment were used to determine the 95% confidence intervals as these two nutrient fractions performed best in the regression model.

**Table 3:** **Pearson correlation matrix for the nutrient fractions (n=50)**

		PO_4_	Total P	NO_3_ + NO_2_	Total N	Total P sediment
**Total P**	Pearson correlation	.641*				
	Sig. (2-tailed)	.000				
**NO**_**3**_ **+ NO**_**2**_	Pearson correlation	-.067	-.024			
	Sig. (2-tailed)	.644	.868			
**Total N**	Pearson correlation	.017	.146	.483*		
	Sig. (2-tailed)	.904	.311	.000		
**Total P sediment**	Pearson correlation	.141	.479*	-.023	-.055	
	Sig. (2-tailed)	.327	.000	.872	.703	
**Total N sediment**	Pearson correlation	-.010	.270	-.115	.191	.455*
	Sig. (2-tailed)	.946	.058	.425	.184	.001

Significant correlations (α=0.05) are indicated by *.

**Figure 3:**
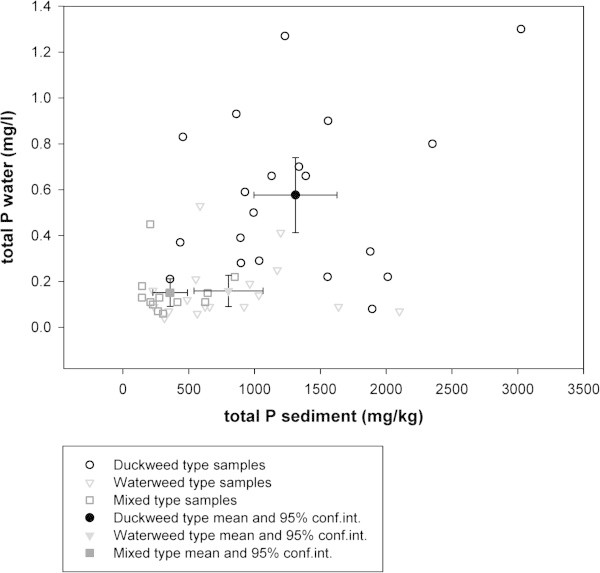
**Scatter biplot for total P in water and sediment, measured in June 2007.** Individual samples (n=50) are shown together with the mean and 95% confidence interval for each of the three vegetation types.

Figure 3 shows that the 95% confidence intervals for total P in water and sediment are rather separated for the three vegetation types, even though there is some overlap in the locations of individual ditches. The ANOSIM resulted in significant group separation between all vegetation types (ANOSIM, R=0.285, mean rank within= 496.1, mean rank between= 670.5, p<0.001). Each vegetation type belonged to an inherent 'group’ with p-values for each comparison of two vegetation types ranging from 0.000 to 0.020. The Mixed type mainly occurred at total P sediment concentrations between 236 and 482 mg/kg (respectively lower and upper boundary of 95% confidence interval) while the Waterweed type is mainly found at higher concentrations (between 551 and 1052 mg/kg). The 95% confidence interval of the Duckweed type shows some overlap with that of the Waterweed type but is mainly restricted to the highest values, ranging from 1008 to1614 mg/kg. The Duckweed type occurs at higher total P concentrations in water (between 0.42 and 0.73 mg/L) than a submerged vegetation (Waterweed or Mixed type). Both the Waterweed and Mixed type are mainly found at values between 0.10 and 0.22 mg/L.

## Discussion

### Nutrients and occurrence of monocultures

Results show that P in both water and sediment contribute to explaining the occurrence of monocultures in shallow aquatic systems. Although the importance of both sediment and water nutrient levels has been shown for lakes (Squires and Lesack 2003; James et al. 2005), the importance of both compartments has not been shown before for shallow aquatic systems. Total P in water only differed between ditches dominated by duckweed and ditches with one of the two submerged vegetation types, while sediment P concentrations especially play an important role in distinguishing the Waterweed type from the Mixed type. Although minerals such as iron and manganese may also play a role in macrophyte growth (Steinberg 1946), results of the present study confirm the importance of P found for lakes, streams and in some drainage ditch studies (Portielje and Roijackers 1995; Thiébaut and Muller 1998; Hilt et al. 2006; van Liere et al. 2007). The role of nutrients in both water and sediment is in line with the large influence of soil-water interactions found in shallow aquatic systems (Herzon and Helenius 2008). Although limitation by both N and P may occur frequently in aquatic systems (Elser et al. 2007), N limitation does not seem to occur in the present study since N fractions showed weak relations with the vegetation types. The continuous high input of N from agricultural fields into surface water (Lamers et al. 2002) may cause a non-limiting availability, while high uptake rates may cause low measured concentrations in water.

The three vegetation types occur in distinctive ranges of nutrient concentrations. The occurrence of the two monocultures is related to higher P concentrations in water (for the Duckweed type) and sediment (for both the Duckweed and Waterweed type) compared to the Mixed type. Chambers (1987) also found that increased nutrient concentrations in the sediment led to a shift from rosette and bottom dwelling species like *Chara* sp. to canopy forming species like *E. canadensis* Michx. *E. nuttallii* is also able to survive under oligo-mesotrophic conditions (Nagasaka 2004) though it will likely not develop into a monoculture under these conditions due to slower uptake of nutrients by roots and therefore slower growth (Angelstein and Schubert 2008; Angelstein et al. 2009).The transition from a diverse submerged vegetation to *E. nuttallii* dominance occurs only at higher sediment P concentrations at which *E. nuttallii* can outcompete other submerged plants (Nichols and Shaw 1986). The capability of *E. nuttallii* to become dominant is illustrated by the higher Simpson’s index (indicating a higher degree of dominance) and lower number of red list species in the Waterweed type, compared to the more heterogeneous Mixed type. The lack of a clear relation between higher water P concentrations and the occurrence of the Waterweed and Mixed type might result from the preferred uptake of nutrients from the sediment by submerged plants, irrespective of the nutrient concentrations in water (Carignan and Kalff 1980). The relation between the Duckweed type and higher water P concentrations can be expected as duckweed only has access to the water phase as a nutrient source while simultaneously having the primacy for light, enabling it to limit submerged plant growth at high water nutrient levels (Portielje and Roijackers 1995; Scheffer et al. 2003; van Liere et al. 2007; Herzon and Helenius 2008). The positive relation with sediment P concentrations is likely caused by the release of P from the anoxic but nutrient rich sediments into the water phase through which it becomes available for duckweed (Janse and Van Puijenbroek 1998).

The Mixed type is mostly found in ditches with sediment that can be classified as oligo- to mesotrophic, while both the Waterweed and Duckweed type are found in ditches with sediments classified as eutrophic (Bloemendaal and Roelofs 1988). The lower range of total P concentrations in water at which duckweed dominance was found in this study is comparable to the model predictions by van Liere et al. (2007) who indicated that a shift from submerged vegetation to duckweed dominance in ditches would occur at water P concentrations between 0.2 and 0.4 mg/L. Most ditches with duckweed dominance are found at higher total water P concentrations.

### Biomass

No differences in total biomass were found between the vegetation types. This is in contrast with for instance Bloemendaal and Roelofs (1988), who describe an optimum curve for the relation between water P levels and macrophyte biomass. They describe that the highest biomass (up to 1200 g DW/m^2^) is found at meso- to eutrophic conditions at which a submerged vegetation produces biomass throughout the whole water column. At eutrophic to hypertrophic conditions, when only duckweed dominates, the total biomass is lower (up to 200 g DW/m^2^) as production is only located at the top of the water column. In the present study the total biomass of the Mixed type confirms the ranges described for mesotrophic systems with a mixed vegetation or with *Potamogeton* species (Forest 1977; van Wijk 1988) and also the total biomass in the Duckweed type is in accordance with previous studies (Janse and Van Puijenbroek 1998; Driever et al. 2005). Interestingly, the total biomass of the Waterweed type is approximately 5 times lower compared to other studies by Di Nino et al. (2005) and Ozimek et al. (1990) who report values from 500 to more than 800 g DW/m^2^. A possible explanation for this relatively low *E. nuttallii* biomass in drainage ditches might be the limited depth of most drainage ditches (depths are comparable between the Waterweed and Mixed type). The high biomass reported in literature (Ozimek et al. (1990) and Pokorný et al. (1984)) for *Elodea* species were obtained in deeper water systems. Due to the limited depth, *E. nuttallii* might not be able to use additional available nutrients to produce more biomass when all available space is already filled. Thus, if all space in the water column is already used, additional available nutrients will not lead to more biomass. A second and alternative explanation could be that increased P-uptake efficiency of *E. nuttallii* at higher P availability led to faster growth (Garbey et al. 2004; James et al. 2006) and increased P concentrations in plant tissue (Sterner and Elser 2002; Garbey et al. 2004). This higher uptake efficiency at higher P availability may consequently be reflected in higher tissue P concentrations rather than in higher total biomass. The stored P may be used to produce vegetative reproductive organs or to maintain growth if nutrient concentrations in the water decrease. Garbey et al. (2004) showed that *Callitriche platycarpa* Kütz. and *C. hamulata* Kütz. ex Koch displayed lower nutrient use efficiencies and tissue P content at increased nutrient availability compared to *E. nuttallii*. Several other species characteristic for a more diverse vegetation such as *Chara vulgaris* L., *Potamogeton zosteriformis* Fernald (Hough et al. 1989), *Potamogeton lucens* L. (Mazej and Germ 2008) and *Myriophyllum alterniflorum* DC. (Fernández-Aláez et al. 1999) also show relatively low tissue P concentrations. If these species are less able to profit from higher nutrient availability by additional growth or storage (Demars and Edwards 2007) they may be overgrown by species like *E. nuttallii*. This way higher P concentrations may result in the development of the Waterweed type at comparable total biomass. However, for many of the mentioned species from more diverse vegetation little is known about the plasticity in nutrient uptake efficiency. This might be caused by a lack of studies on this subject, though several of these species might hardly be found under highly eutrophic conditions. A possible third explanation for the low biomass of the Waterweed type could be the effect of mowing which is common in the Netherlands to secure the drainage function of highly productive ditches (Peeters 2005). Di Nino et al. (2005) found a maximum standing stock of *E. nuttallii* in a stream of 822 g DW/m^2^ without cutting and 180 g DW/m^2^ with cutting early in the growing season. However, *E. nuttallii* will likely remain dominant in these disturbed systems due to its fast regrowth and regeneration from stem fragments (Di Nino et al. 2005).

### Implications for water quality management

Considering the negative effects of dominance by both duckweed and *E. nuttallii* on ecosystem functioning (Janse and Van Puijenbroek 1998; Di Nino et al. 2005) it seems necessary for water managers to lower both water and sediment P levels in drainage ditches with a monoculture to values at which the Mixed type was found in this study. However, to cause a shift towards the Mixed type even lower nutrient levels might be needed to overcome the possible resilience of the Duckweed type (Scheffer et al. 2001). Rigorous additional measures such as removal of the propagule bank of duckweed or reintroduction of previously present submerged species might further support reestablishment of the Mixed type (van Zuidam et al. 2012; Hilt et al. 2006).

The focus of present water quality legislation in the Netherlands is only on nutrient levels in water with targets for total P in water being around 0.2 mg P/L (Evers et al. 2007). Considering the present study, lowering P levels of inflowing water to concentrations below 0.2 mg/L will probably be insufficient to prevent duckweed dominance when sediment P concentrations are still higher than 1000 mg/kg. Exchange of P from the sediment to the water will likely elevate the P concentrations in water (Roelofs 1991), supporting duckweed dominance, thereby raising the need for additional removal of P rich sediments. To suppress excessive growth of *E. nuttallii* and promote development of the Mixed type, a reduction of P in sediment to concentrations below 500 mg/kg is likely needed. It seems useful to include targets for sediment P concentrations in current water quality legislation, although negative effects of dredging should be taken into account when considering large scale sediment removal. Among possible negative effects are loss of aquatic fauna (Twisk et al. 2000), decreased nutrient removal by benthic biota and increased nutrient fluxes from newly exposed, nutrient rich layers (Smith and Pappas 2007).

Other limiting factors for vegetation recovery in drainage ditches might be the presence of herbicides like atrazine which causes photosynthetic inhibition (Graymore et al. 2001), heavy metals which accumulate in plant material (Martins et al. 2011) and frequent mowing causing reduced growth and reproduction (van Zuidam and Peeters 2012). Even though reduced nutrient concentrations might create favourable conditions for restoration of the Mixed type, frequent mowing might still cause persistence of the Waterweed type as *E. nuttallii* is capable of showing fast regeneration and spreading after disturbance (Barrat-Segretain et al. 1998). Fast recovery of *E. nuttallii* may cause lowering of nutrient availability in the water though, possibly limiting the possibilities for duckweed to become dominant. This way *E. nuttallii* can have a positive effect as pioneer species in disturbed conditions. However, since Peeters (2005) showed that the highest species richness in ditches was found at intermediate mowing frequencies, lowering mowing frequency as an additional measure might be essential to further stimulate recolonisation by other submerged species.

## Electronic supplementary material

Additional file 1: **Observed plant species and nr. of observations.** (DOC 58 KB)
