# Supplementary material for: Occurrence of macrophyte monocultures in drainage ditches relates to phosphorus in both sediment and water
Source: Springerplus. 2013 Oct 25;2:564. doi: 10.1186/2193-1801-2-564 (PMC3825067; doi:10.1186/2193-1801-2-564)
Supplement: Supplementary file 1 — Additional file 1: Observed plant species and nr. of observations. (DOC 58 KB) [file 40064_2013_641_MOESM1_ESM.doc]

**Additional file 1. Observed plant species and nr. of observations**

| **Species name** | **Total nr. of observations** | **Highest observed coverage (Tansley class)** |
| --- | --- | --- |
| Filamentous algae | 62 | 9 |
| *Azolla filiculoides* Lam. | 30 | 9 |
| *Fontinalis antipyretica* Hedw. | 8 | 5 |
| *Callitriche* sp. L. | 26 | 7 |
| *Ceratophyllum demersum* L. | 64 | 9 |
| *Chara* sp. L. | 17 | 8 |
| *Egeria densa* Planch. | 2 | 2 |
| *Elodea canadensis* Michx. | 11 | 9 |
| *Elodea nuttallii* (Planch.) H. St. John | 86 | 9 |
| *Groenlandia densa* L. | 1 | 1 |
| *Hottonia palustris* L. | 2 | 2 |
| *Hydrocharis morsus-ranae* L. | 38 | 8 |
| *Lemna gibba* L. | 54 | 9 |
| *Lemna minor* L. | 111 | 9 |
| *Lemna minuta* Kunth. | 30 | 9 |
| *Lemna trisulca* L. | 75 | 9 |
| *Myriophyllum alterniflorum* DC. | 1 | 6 |
| *Myriophyllum spicatum* L. | 19 | 9 |
| *Myriophyllum verticillatum* L. | 4 | 2 |
| *Nitella* sp. C. Agardh. | 12 | 9 |
| *Nuphar lutea* (L.) Sm. | 9 | 5 |
| *Nymphoides peltata* (S.G.Gmel.) Kuntze | 8 | 8 |
| *Nymphaea alba* L. | 4 | 7 |
| *Potamogeton acutifolius* Link | 3 | 3 |
| *Potamogeton berchtoldii* Fieber | 1 | 5 |
| *Potamogeton compressus* L. | 3 | 6 |
| *Potamogeton crispus* L. | 9 | 5 |
| *Potamogeton lucens* L. | 8 | 8 |
| *Potamogeton mucronatus* Sonder | 2 | 2 |
| *Potamogeton natans* L. | 11 | 6 |
| *Potamogeton obtusifolius* Mert. & Koch | 9 | 6 |
| *Potamogeton pectinatus* L. | 6 | 5 |
| *Potamogeton perfoliatus* L. | 3 | 2 |
| *Potamogeton pusillus* L. | 1 | 2 |
| *Potamogeton trichoides* Cham. & Schltdl. | 18 | 7 |
| *Ranunculus circinatus* Sibth. | 8 | 6 |
| *Ranunculus* sp. L. | 7 | 2 |
| *Riccia fluitans* L. | 16 | 7 |
| *Ricciocarpos natans* L. | 2 | 7 |
| *Spirodela polyrhiza (*L.) Schleid. | 81 | 6 |
| *Stratiotes aloides* L. | 5 | 9 |
| *Utricularia* sp. L. | 4 | 8 |
| *Wolffia arrhiza (*L.) Horkel ex Wimm. | 19 | 8 |
| *Zannichellia palustris* L. | 6 | 5 |
